# Crucial Involvement of Heme Biosynthesis in Vegetative Growth, Development, Stress Response, and Fungicide Sensitivity of *Fusarium graminearum*

**DOI:** 10.3390/ijms25105268

**Published:** 2024-05-12

**Authors:** Jin Wang, Yingying Cao, Dongya Shi, Zhihui Zhang, Xin Li, Changjun Chen

**Affiliations:** College of Plant Protection, Nanjing Agricultural University, Nanjing 210095, China; 2019202058@njau.edu.cn (J.W.); 2021102106@njau.edu.cn (Y.C.); 2018202051@njau.edu.cn (D.S.); 2021202057@njau.edu.cn (Z.Z.); 2020102109@stu.njau.edu.cn (X.L.)

**Keywords:** *Fusarium graminearum*, heme, growth, conidiation, stress response, β-oxidation, FgCyp51, virulence

## Abstract

Heme biosynthesis is a highly conserved pathway from bacteria to higher animals. Heme, which serves as a prosthetic group for various enzymes involved in multiple biochemical processes, is essential in almost all species, making heme homeostasis vital for life. However, studies on the biological functions of heme in filamentous fungi are scarce. In this study, we investigated the role of heme in *Fusarium graminearum*. A mutant lacking the rate-limiting enzymes in heme synthesis, coproporphyrinogen III oxidase (Cpo) or ferrochelatase (Fc), was constructed using a homologous recombination strategy. The results showed that the absence of these enzymes was lethal to *F. graminearum*, but the growth defect could be rescued by the addition of hemin, so we carried out further studies with the help of hemin. The results demonstrated that heme was required for the activity of FgCyp51, and its absence increased the sensitivity to tebuconazole and led to the upregulation of *FgCYP51* in *F. graminearum*. Additionally, heme plays an indispensable role in the life cycle of *F. graminearum*, which is essential for vegetative growth, conidiation, external stress response (especially oxidative stress), lipid accumulation, fatty acid β-oxidation, autophagy, and virulence.

## 1. Introduction

Fusarium head blight (FHB), caused by the *Fusarium graminearum* species complex, is a devastating fungal disease of wheat worldwide [1]. FHB not only reduces grain yields, but the mycotoxin deoxynivalenol (DON) produced by the pathogen also threatens the safety and health of humans and livestock [2,3]. Due to the lack of commercially available resistant or immune varieties, FHB is mainly controlled with fungicides [4]. Sterol synthesis demethylase inhibitors (DMIs), such as tebuconazole, propiconazole, epoxiconazole, and prochloraz, which target lanosterol 14-alpha-demethylase (Cyp51, homology of Erg11, ergosterol-related genes 11), are widely used for FHB control in the field because they have a good control effect and inhibit DON production [5]. However, strains resistant to DMIs have been detected in the field [6,7].

Sterols are essential components of the cytomembrane to maintain cell integrity and fluidity [8,9,10]. Sterol homeostasis is critical for organismal activity. To avoid adverse consequences of sterol depletion, sterol regulatory element-binding proteins (SREBP) and sterol uptake control proteins (Upc2) are two sterol regulators that sense intracellular sterol levels and regulate sterol synthesis [11,12,13,14,15,16,17]. However, the loss of homologous genes of SREBP or Upc2 in *F. graminearum* has been shown to be unrelated to the sensitivity to DMIs. Nevertheless, a novel mechanism has been discovered in which a transcription factor regulates the ergosterol synthesis and the sensitivity to DMIs by regulating the expression of FgCyp51 once challenged with DMIs [18].

Structural analysis of target proteins has been used in the design of new drugs in recent years. Analysis of the crystal structure of the *Saccharomyces cerevisiae* Erg11 protein revealed that a heme molecule is bound to its center, where the heme acts as a cofactor [19]. Moreover, heme enzymes also play roles in oxygen transport, electron transfer, fatty acid synthesis, and the oxidative system [20,21,22,23,24]. The synthesis pathway is preserved through evolution and is highly conserved, from bacteria to higher animals. Heme, a member of the porphyrin family, consists mainly of a porphyrin ring and a central Fe atom. The Fe atom is important for enzyme activity and is involved in processes such as redox reactions, gas sensing, electron transport, cell cycle progression and proliferation, mitochondrial autophagy, and apoptosis [25,26,27]. Besides, heme homeostasis is important for the stability of the intracellular environment. For example, disruption of heme metabolism leads to the generation of hydroxyl radicals via the Fenton reaction, which damage DNA, membrane lipids, proteins, etc. [28,29,30], so its synthesis is strictly regulated. Coproporphyrinogen III oxidase (Cpo) and ferrochelatase (Fc) are two rate-limiting enzymes that regulate the production of heme [31]. Although the enzymes in this pathway are relatively conserved, Cpo or Fc show considerable divergence across species [32].

However, the function of heme in filamentous fungi has not been demonstrated directly. The aim of this research is to understand the specific role of heme in *Fusarium graminearum*, including its function in DMI sensitivity, pathogenicity, and biochemical functions related to growth and development. In this study, we confirmed that heme is involved in the function of FgCyp51 in *F. graminearum* and regulates the sensitivity to DMIs. Furthermore, heme affects the vegetative growth, conidiation, virulence, stress response, lipid accumulation, fatty acid β-oxidation, and autophagy of the pathogen.

## 2. Results

### 2.1. The Identification and Localization of FgCpo and FgFc

**Figure 1:**
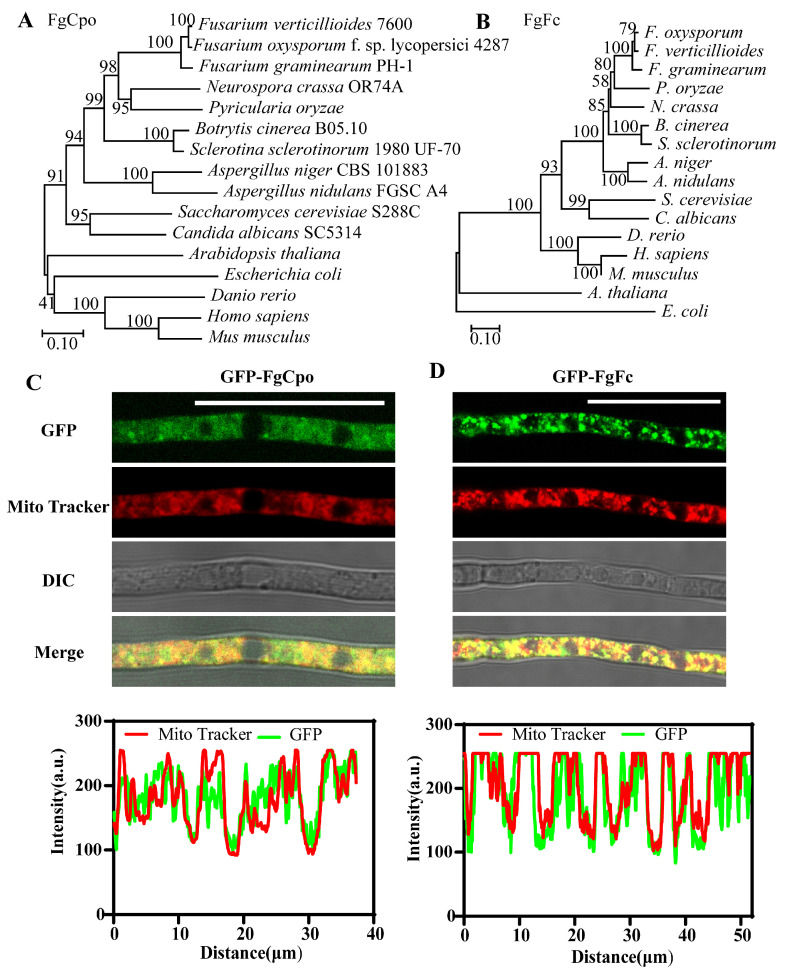
Identification of Cpo and Fc in *Fusarium graminearum*. Phylogenetic analysis of Cpo (**A**) and (**B**) Fc among serval species. The phylogenetic tree was constructed based on the amino acid sequence of Cpo/Fc of the species in the figure by MEGA 11 with the neighbor-joining method. Localization of FgCpo (**C**) and FgFc (**D**) and the co-localization analysis. Bar = 25 μm.

Coproporphyrinogen III oxidase and ferrochelatase genes (designated *FgCPO* and *FgFC*) were retrieved with a BLASTp search of the NCBI database using *S. cerevisiae* Cpo (YDR044W) and Fc (YOR176W) as a query, respectively. And the protein sequences of Cpo or Fc from different species were blasted with FgCpo or FgFc, respectively. The phylogenetic tree was constructed with the protein sequence blasted with Cpo or Fc from different species by MEGA 11 with the neighbor-joining method. And we compared Cpo and Fc from bacteria to humans and found that FgCpo or FgFc shared high homology in *Fusarium* and varied among species (Figure 1A,B). To determine the intracellular localization of FgCpo and FgFc in the cell, a vector fused with GFP at the N-terminal of FgCpo or FgFc was induced into the wild-type strain PH-1 by protoplast transformation, respectively. Fresh mycelia of the resulting transformants stained with Mito Tracker (a mitochondrial dye, Thermo Fisher Scientific, Waltham, MA, USA) for 30 min at 37 °C were observed under the laser confocal scanning microscope (Leica TCS SP8, Berlin, Germany). The fluorescence signal of the Mito Tracker was excited by a 561 nm excitation light, and the emission light wavelength was set between 582 and 754 nm. Images were analyzed for co-localization (ImageJ, v. 1.51j8, Bethesda, MD, USA). As shown in Figure 1C,D, the fluorescence signal of GFP and Mito Tracker largely overlapped, indicating that the FgCpo and FgFc were mainly localized in the mitochondria.

### 2.2. Heme Deficiency Is Fatal for F. graminearum

**Figure 2:**
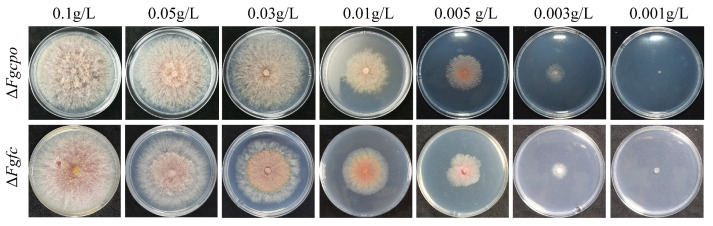
Colony morphology of Δ*Fgcpo* and Δ*Fgfc* on serial concentrations of hemin plates cultured at 25 °C for 4 d.

When we tried to knockout *FgCPO* or *FgFC* with a double homologous exchange strategy, partial transformants died after being transferred to new CM plates, and the other parts were negative transformants. Positive transformants were obtained by adding hemin (heme Fe^3+^), an analogue of heme, into the medium. The resulting transformants were validated by PCR and Southern blot (Figure S1). Then, mycelial discs of mutant Δ*Fgcpo* or Δ*Fgfc* were transferred onto CM plates supplemented with various concentrations of hemin. As shown in Figure 2, the two mutants recovered growth on plates with a higher heme concentration (0.1 g/L), but growth defects became apparent with decreasing heme concentration, and aerial hyphae gradually disappeared. Mutants failed to form colonies on plates containing 0.001 g/L of heme. These results confirmed that the defect in heme biosynthesis was lethal to *F. graminearum*.

### 2.3. Heme Deficiency Impairs the Activity of FgCyp51

**Figure 3:**
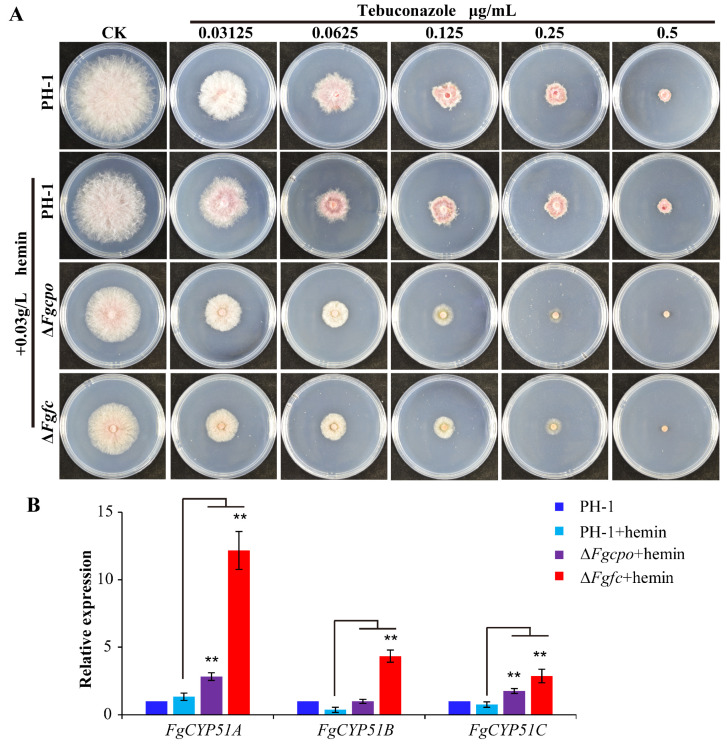
EC_50_ for tebuconazole and the relative expression of *FgCYP51*. (**A**) The colony morphology of the mutants on CM plates supplemented with tebuconazole. (**B**) The relative expressions of *FgCYP51A/B/C* in the parent strain and mutants. The data shown are the mean ± SD. The stars above the bars denote that they are not significantly different based on a one-way ANOVA followed by Dunnett’s T3 test (** *p* < 0.05).

To assess the effect of heme on the function of Cyp51 in *F. graminearum*, the sensitivities of Δ*Fgcpo* and Δ*Fgfc* to tebuconazole were determined. Both EC_50_ and MIC (minimal inhibitory concentration) of the two deleted mutants to tebuconazole were significantly decreased compared to the parent strain PH-1 treated with hemin (Figure 3A and Table 1). Additionally, the parent strain showed reduced sensitivity to tebuconazole after the addition of hemin. Since the expressions of *FgCYP51* are highly induced when the activities of FgCyp51 are inhibited by DMIs [18], the relative expressions of *FgCYP51A/B/C* (three homology genes of *CYP51* in *F. graminearum*) in the mutants were tested. As shown in Figure 3B, the expressions of *FgCYP51A/B/C* in Δ*Fgcpo* and Δ*Fgfc* were all significantly upregulated compared to PH-1 + hemin treatment. And the addition of hemin did not affect the expression of *FgCYP51A/B/C* in the wild-type strain PH-1. These results indicated that heme deficiency increased the sensitivity to tebuconazole and damaged the activity of FgCyp51 in *F. graminearum*.

### 2.4. Heme Deficiency Impairs the Growth Rate, Asexual Reproduction, and Germination of Conidia

**Figure 4:**
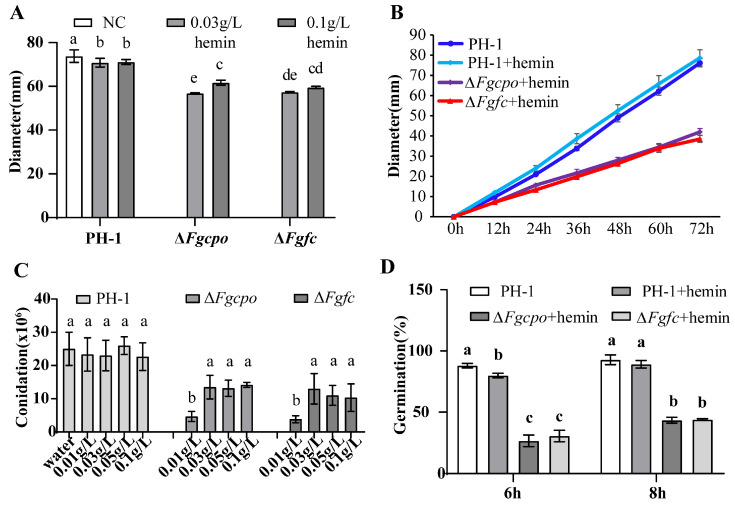
Growth diameter on different mediums, conidiation, and germination of each strain. (**A**) The colony diameter on CM medium supplemented with 0.03/0.1 g/L hemin at 25 °C for 3 d. (**B**) The growth rate curve of each strain for 3 d of incubation at 25 °C on CM medium supplemented with 0.03 g/L hemin heme. The diameter was recorded every 12 h. (**C**) The conidia production was incubated in mung bean liquid medium containing a serial concentration of hemin at 25 °C for 7 d. (**D**) The germinated conidia rate on water agar plates at 25 °C for 6 h/8 h. The data shown are the mean ± SD. The same letters above the bars denote that they are not significantly different based on a one-way ANOVA followed by a Fisher’s LSD test (*p* = 0.05).

The colony diameters of Δ*Fgcpo* and Δ*Fgfc* on CM medium supplemented with 0.03/0.1 g/L hemin were significantly decreased compared to the parental strain (Figure 4A). In addition, the growth rate decreased in the mutant compared to the parental strain (Figure 4B), indicating that the deficiency of heme damaged the growth of *F. graminearum.*

Besides, we determined the conidiation of the strains. After 7 d of incubation in MBB, the conidia of each strain were collected and counted. A few conidia were produced in the deleted mutants without the addition of hemin. Upon addition of hemin to MBB, the mutants regained the ability to sporulate, and sporulation was higher in medium with a high concentration (0.03 g/L) compared to a lower concentration (0.01 g/L) (Figure 4C). However, higher concentrations of hemin (0.05 or 0.1 g/L) did not further increase conidiation. Compared to the wild type, both Δ*Fgcpo* and Δ*Fgfc* mutants showed a reduction in conidiation. These results indicated the importance of heme in the asexual reproduction of *F. graminearum*. In addition, the germination of conidia was affected in the Δ*Fgcpo* and Δ*Fgfc mutants*. The conidia germination rate of PH-1 was 88% after 6 h of incubation on a water agar plate and 93% after 8 h of incubation. In contrast, the conidia germination rates of Δ*Fgcpo* and Δ*Fgfc* remained below 50% after 8 h (Figure 4D).

### 2.5. Heme Deficiency Damages the Full Virulence of F. graminearum

**Figure 5:**
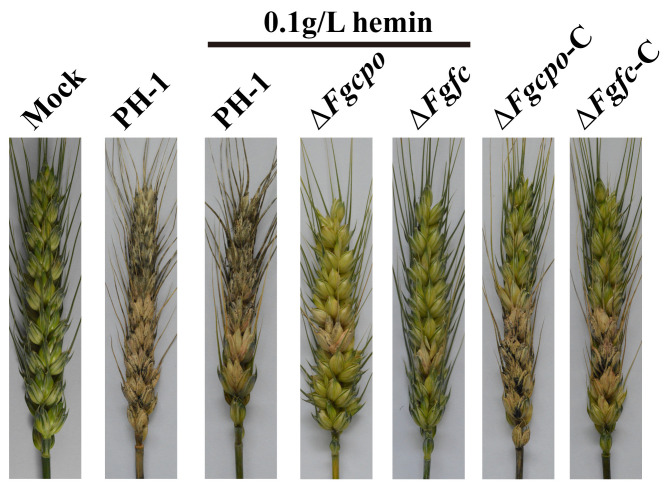
Disease scabs on flowering wheat heads after being inoculated for 21 d in the field.

To clarify the effect of heme on virulence, conidia were inoculated onto the flowering wheat heads. Considering the defective germination of Δ*Fgcpo* and Δ*Fgfc*, hemin (final concentration 0.1 g/L) was added to the conidia suspension to promote the colonization of spores on wheat heads. As shown in Figure 5, the conidia of two mutants were able to colonize on the wheat head with the help of hemin, but the scab caused by the mutant did not expand like that of the wild-type strain, indicating a significant reduction in virulence to the wheat head.

### 2.6. Heme Deficiency Affects Conidia Morphology

**Figure 6:**
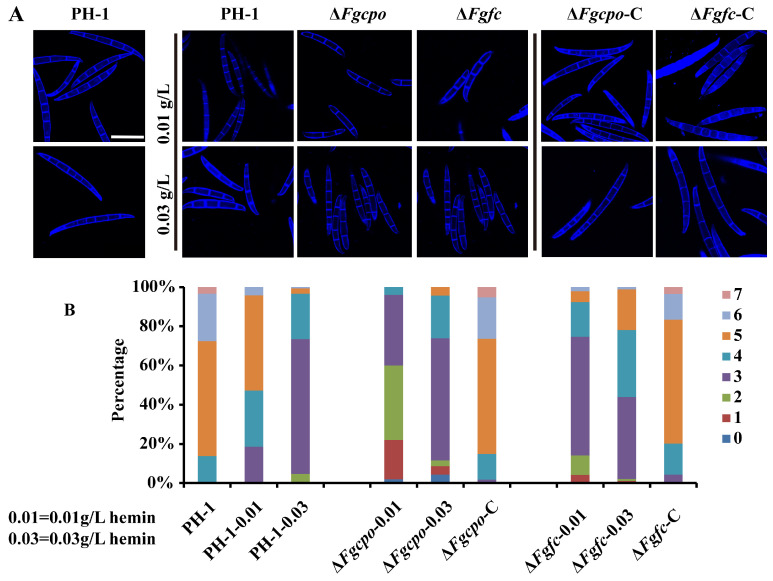
Conidia morphology. (**A**) The conidia of each strain were stained with calcofluor white and observed under a laser confocal fluorescence microscope. Bar = 50 μm. (**B**) Percentage of conidia (*n* = 200) with 0–7 septum numbers of each strain.

When measuring the conidial production, we observed a significant difference in the morphology of the conidia of Δ*Fgcpo* and Δ*Fgfc* compared to the wild-type strain. To further visualize conidial morphology, conidia were stained with Calcofluor White (CFW, Sigma-Aldrich, Merck, Darmstadt, Germany). The fluorescence signal of CFW was excited by a 405 nm excitation light, and the emission light wavelength was set between 410 and 476 nm. The conidia of the mutants had significantly fewer septa than those of PH-1 under the same conditions (Figure 6). The number of septa in 200 conidia was counted and showed that the majority of 7-day-old wild-type conidia had five to seven septa, whereas the majority of mutant conidia had only two to three septa (Figure 6B). The number of septa in conidia produced under low-hemin conditions was significantly reduced. Although the number of septa in conidia on high hemin media increased, it was still less than in the wild-type strain. However, the additional hemin reduced the number of septa in the conidia of PH-1. These results suggested that heme deficiency affected the morphology of conidia.

### 2.7. Heme Deficiency Impairs the Sensitivity to Stress Factors

**Figure 7:**
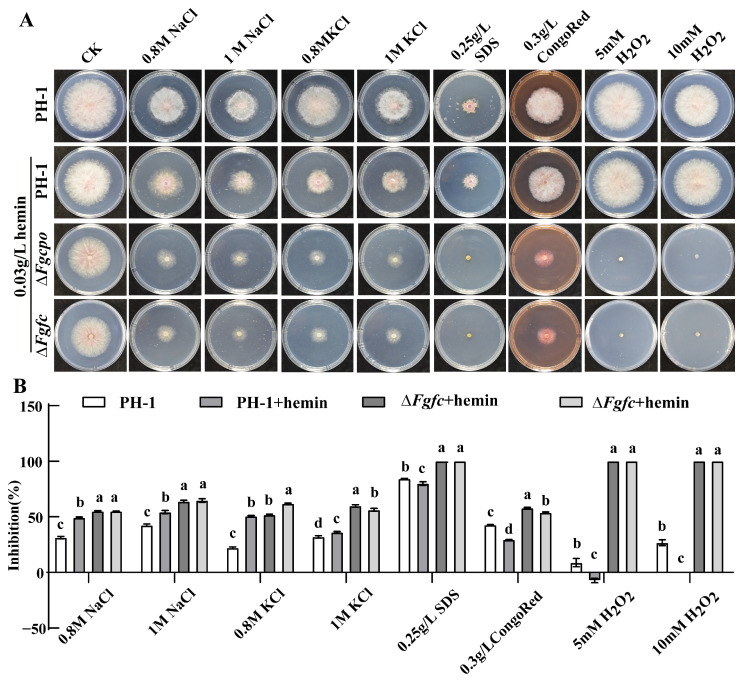
Sensitivity to various stressors for each strain. (**A**) Colonies morphology on CM plates containing osmotic stress, cell membrane stress, and cell wall stress oxidative stress factors incubated at 25 °C for 3 d. (**B**) Inhibition rate of mycelial growth by different stress factors. The data shown are the mean ± SD. The same letters above the bars denote that they are not significantly different based on a one-way ANOVA followed by a Fisher’s LSD test (*p* = 0.05).

To determine whether heme affects tolerance to oxidative stress, osmotic stress, cell wall stress, and cell membrane stress, we determined the inhibition of growth in all strains treated with 1.2/0.8 M NaCl, 1.2/0.8 M KCl, 0.25 g/L SDS, 0.3 g/L Congo Red, or 5/10 mM H_2_O_2_. The results (Figure 7) showed that the addition of hemin would enhance the resistance of the wild-type strain to cell wall stressors, cell membrane stressors, and oxidative stressors. In contrast, the two mutants, namely, Δ*Fgcpo* and Δ*Fgfc*, became more sensitive to all stressors. Although hemin restored growth and sporulation in the mutants, it appeared to lose its function when exposed to oxidative stress. Moreover, 5 mM H_2_O_2_ did not inhibit the growth of PH-1 at all but completely inhibited the growth of Δ*Fgcpo* and Δ*Fgfc*. This result demonstrated that heme plays an important role in resisting external stress.

### 2.8. Heme Deficiency Affects the Lipid Drop and Glycerol Accumulation

**Figure 8:**
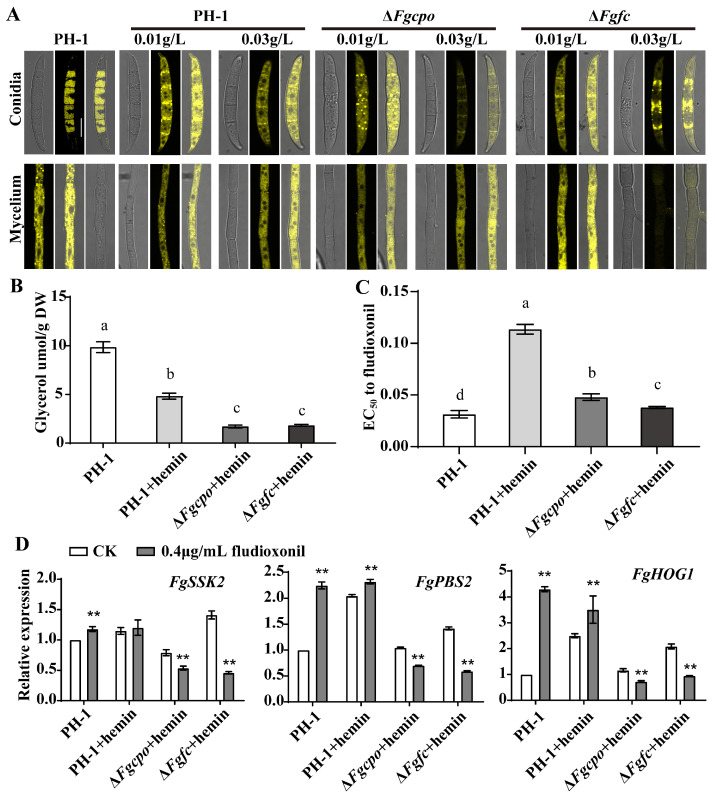
Lipid droplets, glycerol accumulation, and gene expression of the HOG cascade pathway. (**A**) Lipid droplets in conidia and mycelia were stained with Nile Red and observed under a laser confocal fluorescence microscope. Bar = 10 μm. (**B**) The concentration of glycerol in each strain. (**C**) EC_50_ values for fludioxonil in the mutants. (**D**) Relative expressions of three key genes in the HOG-cascade pathway of each strain. Mycelia were incubated in CM liquid medium containing 0.3 g/L hemin at 25 °C for 3 d. 0.4 μg/mL fludioxonil was added to the medium for 6 h before collection. The data shown in B and C are the mean ± SD. The same letters above the bars denote that they are not significantly different based on a one-way ANOVA followed by a Fisher’s LSD test (*p* = 0.05). The stars above the bars denote that they are not significantly different based on a one-way ANOVA followed by Dunnett’s T3 test (** *p* < 0.05).

To clarify the role of hemin in the accumulation of lipid droplets and glycerol, we observed the lipid droplets in conidia/mycelia stained with Nile Red (Macklin, Shanghai, China) and measured the glycerol concentration in the mycelia. As shown in Figure 8A, a significant reduction in lipid droplets was observed in both the conidia and mycelia of Δ*Fgcpo* and Δ*Fgfc* mutants. Although many fluorescent spots were present, these signals were generated under higher laser power. And the concentration of glycerol was also reduced in the two mutants (Figure 8B). In addition, the two mutants showed increased sensitivity to fludioxonil. However, the extra hemin reduced the sensitivity against fludioxonil in the wild-type strain (Figure 8C, Table 1). We further examined the expression of key genes in the HOG pathway (Figure 8D) and demonstrated that after treatment with fludioxonil, the expression of *FgSSK2/FgPBS2/FgHOG1* was upregulated or unchanged in the wild-type strain, whereas it was significantly downregulated in the mutants. These results suggested that heme deficiency would disturb the regulation of lipid droplet and glycerol accumulation and the modulation of the HOG pathway.

### 2.9. Heme Deficiency Affects Fatty Acid ꞵ-oxidation

**Figure 9:**
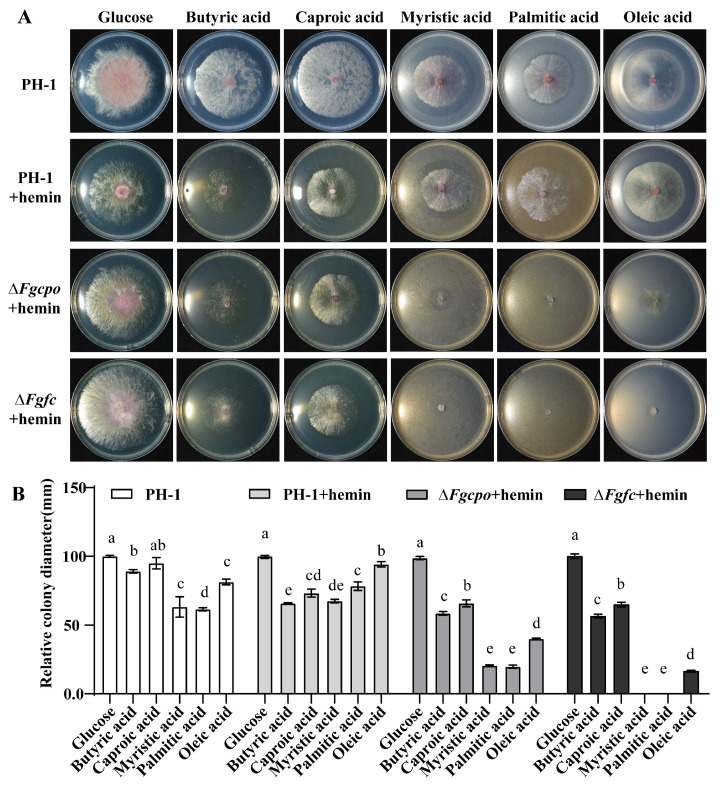
Determination of fatty acid utilization. (**A**) Colony morphology of each strain inoculated on MM-C plates containing 0.03 g/L hemin supplemented with several fatty acids, respectively, at 25 °C for 5 d. (**B**) The relative colony diameter of each strain. Relative diameter means the diameter of each treatment/the diameter of glucose. The data shown are the mean ± SD. The same letters above the bars denote that they are not significantly different based on a one-way ANOVA followed by a Fisher’s LSD test (*p* = 0.05).

To understand whether heme is involved in the utilization of fatty acids, different fatty acids such as butyric acid, caproic acid, myristic acid, palmitic acid, or oleic acid were added separately into the MM-C medium as the sole carbon source, and the growth of PH-1 and the two mutants on these media was determined. The results (Figure 9) showed that the relative colony diameters of Δ*Fgcpo* and Δ*Fgfc* mutants on fatty acid media, especially those containing long-chain fatty acids, were significantly reduced, indicating a marked decrease in the ability of the mutants to utilize the fatty acids. This result suggested that heme deficiency affected the β-oxidation of fatty acids.

### 2.10. Heme Negatively Regulates Autophagy

**Figure 10:**
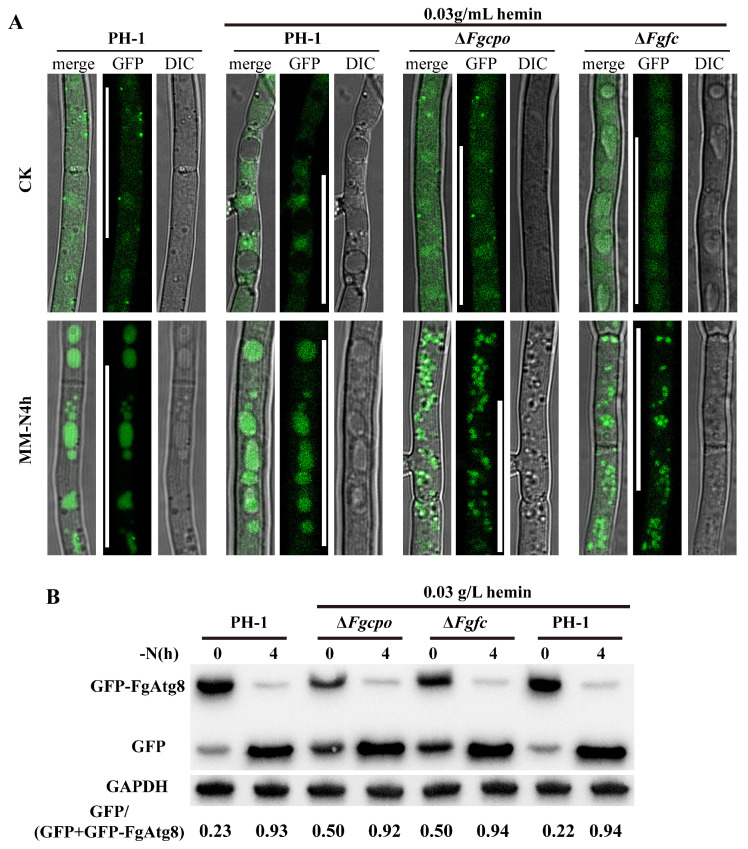
Autophagy detection. (**A**) GFP-FgAtg8 localization in the mutants. Mycelia were incubated in CM liquid medium for 24 h and then shifted to MM-N liquid medium for 4 h at 25 °C. Bar = 25 μm. (**B**) The degradation of GFP-FgAtg8 was detected by Western blot. The data at the bottom represent the percentage of degraded GFP-FgAtg8.

Since heme deficiency affects the metabolism of several pathways and cellular autophagy is important for maintaining cellular homeostasis [33,34,35], the autophagy level was determined by observing the localization of GFP-FgAtg8 as well as its degradation detected by Western Blot. First, the GFP signal in the wild-type strain was mainly localized to the cytoplasm in nutrient-rich media, but a large amount of GFP signal was localized to vacuoles in the two mutants Δ*Fgcpo* and Δ*Fgfc* under the same condition (Figure 10A). Then, the degradation of GFP-FgAtg8 was detected by Western blot. The degradation rate of GFP-FgAtg8 in the wild-type strain was about 23%, while the two mutants showed a higher degradation rate (52% and 50%) (Figure 10B) even under nutrient-rich conditions, indicating activated autophagy in the mutants. Supplemental hemin (0.03 g/L) had no significant effect on autophagy in the wild-type strain. These results demonstrated that heme depletion would induce autophagy.

## 3. Discussion

Heme is essential for the survival of organisms. Heme is the cofactor for a variety of enzymes in organisms. In *F. graminearum*, the absence of heme is lethal. Although heme-deficient mutants could survive with hemin, they exhibited marked growth defects, reduced conidiation, altered spore morphology, attenuated virulence, and reduced lipid droplet accumulation. What is worse, the balance of the redox reaction was disrupted. Besides, the ability to utilize fatty acids was greatly reduced, and β-oxidation capability was impaired. Heme deficiency also caused a decreased activity of FgCyp51 in *F. graminearum*, resulting in increased sensitivity to tebuconazole. In summary, the blocking of the synthesis of heme damaged nutritional growth, sporulation, spore germination, stress defense, virulence, and the activities of FgCyp51 in *F. graminearum*.

Heme homeostasis is essential for the normal life activities of organisms. Previous studies have shown that disruption of heme synthesis impairs growth [31,36]. In *Aspergillus fumigatus*, the absence of SreA increases siderophore biosynthesis and iron uptake, leading to increased heme synthesis, at which point Cpo acts as a rate-limiting factor, causing the accumulation of coproporphyrin Ⅲ and preventing the dangerous level of heme in the cytoplasm [37]. Free heme is toxic to cells by generating ROS, leading to lipid peroxidation, protein degradation, and DNA damage [38,39,40,41,42,43]. And overexpression of *CPO* or *FC* in *Aspergillus niger* results in no or a slight increase in heme production [31]. In zebrafish larvae, impaired heme transport leads to the accumulation of heme in mitochondria, causing a variety of diseases during development and ultimately leading to death [44]. In humans, impaired heme metabolism causes porphyria [45,46]. In this study, we provide evidence that disruption of heme synthesis leads to strain death, suggesting that heme is also essential for the survival of the filamentous fungus *F. graminearum*.

Heme is a double-edged sword for resisting environmental stress in organisms. Ascorbate peroxidase (APX) is a heme enzyme and one of the most abundant peroxidases in plants, which effectively scavenges excessive H_2_O_2_ accumulated in cells [47]. Similarly, application of heme and CO in an aqueous solution alleviated seed germination and seedling growth of rice under salt stress [48]. Heme will be degraded by heme oxygenase to prevent excess heme from toxic oxidative stress [49,50,51]. As shown in Figure 8, after the addition of hemin, 5 mM H_2_O_2_ was lethal to the heme-null strain but had no effect on the wild-type strain. Moreover, excess hemin (heme Fe^3+^) impaired the resistance of PH-1 to osmotic stress but improved the resistance to cell wall stress, cell membrane stress, and oxidative stress.

Heme may affect signaling regulation in *F. graminearum*. It has been reported that heme functions as a versatile signaling molecule that regulates the activities of diverse regulators ranging from transcription factors to MAP kinases [52,53,54,55]. Fludioxonil is considered to block glycerol transport by inhibiting the MAP/histidine kinase in osmotic signal transduction [56]. Genes encoding enzymes involved in glycerol synthesis are regulated by Hog1 [57,58], which also controls the uptake of glycerol [59]. Furthermore, the absence of Hog1 can be partially compensated by an artificial, re-routed Hog1-independent signaling system that causes glycerol accumulation, thereby mediating osmotic adaptation [60]. The data in this study (Figure 8) showed that heme-deficient mutants had reduced glycerol concentration and increased sensitivity to osmotic stress, suggesting that heme indeed regulates glycerol accumulation and subsequently regulates osmotic adaptation. In addition, the mutants showed increased sensitivity to fludioxonil, and the three key genes in the HOG cascade were downregulated and could not be induced by fludioxonil as in the wild-type strain. In the future, the specific mechanisms by which heme regulates the HOG pathway need to be further investigated.

## 4. Materials and Methods

### 4.1. Strain, Medium, Fungicide, and Plasmid

The standard *F. graminearum* strain PH-1 was maintained in our laboratory. The knockout mutants and GFP-tagged strains mentioned in this article were all constructed from PH-1. The complete medium (CM, 0.1% Casamino acid (Solarbio Life Science, Beijing, China), 1% glucose (Sinopharm Chemical Reagent Co., Ltd., Shanghai, China), 0.2% peptone (Solarbio Life Science), 0.1% yeast extract (Thermo Fisher Scientific, Waltham, MA, USA), nitrate salts (6 g NaNO_3_ (Xilong Scientific, Shantou, China), 0.52 g KCl (Sinopharm Chemical Reagent Co., Ltd.), 0.52 g MgSO_4_·7H_2_O (Guangdong Guanghua Sci-Tech Co., Ltd., Shantou, China), 1.52 g KH_2_PO_4_ (Guangdong Guanghua Sci-Tech Co., Ltd.)), 0.01% trace elements(1 L: 22 g ZnSO_4_·7H_2_O, 11 g H_3_BO_3_, 5 g MnCl_2_·4H_2_O, 5 g FeSO_4_·7H_2_O, 1.7 g CoCl_2_·6H_2_O, 1.6 g CuSO_4_·5H_2_O, 0.15 g Na_2_MoO_4_·5H_2_O and 5 g Na_4_EDTA, all purchased from Guangdong Guanghua Sci-Tech Co., Ltd., Shantou, China), 0.01% vitamins (biotin, pyridoxine, thiamine, riboflavin, p-aminobenzoic acid, and nicotinic acid, all purchased from Shanghai Ryon Biological Technology Co., Ltd. (Shanghai, China) and 1 L aqua pura, pH 6.5), minimal medium (MM, 0.05% KCl, 0.2% NaNO_3_, 0.1% KH_2_PO_4_, 0.05% MgSO_4_·7H_2_O, 0.001% FeSO_4_·7H_2_O, 3% sucrose, 1 L aqua pura, 0.2% trace elements (100 mL: 5 g ZnSO_4_·7H_2_O, 5 g citric acid, 0.25 g CuSO_4_·5H_2_O, 1 g Fe(NH_4_)_2_(SO_4_)_2_·6H_2_O) (Macklin, Shanghai, China), pH 7.0), and mung bean broth (MBB, 1 L: 30 g mung beans (purchased from a supermarket) were boiled in boiling tap water for 20 min, filtered with four-layers of gauze). The media used were prepared according to previous studies [61]. The fungicides tebuconazole (97% a.i., Jiangsu Aijin Agrochemical Co., Ltd., Nanjing, China) and fludioxonil (97.9% a.i., Yangnong, Yangzhou, China) were used for fungicide sensitivity determination. Hemin (Maclin, Shanghai, China) was used to culture the mutants. Plasmids pYF11 or pDL2 resistant to ampicillin were used for GFP-tagged strain construction and expressed resistance to G-418 or hygromycin B in fungi. Plasmid pKHT resistant to kanamycin was used for the amplification of the HPH cassette fragment, which expresses hygromycin B resistance in fungi.

### 4.2. Vector Construction and Protoplast Transformation

To explore the function of heme in *F. graminerum*, the deleted mutants of *FgCPO* or *FgFC* were constructed, respectively. The nucleotide sequences of *FgCPO* (Accession number: FGSG_10739) and *FgFC* (Accession number: FGSG_05316) were obtained from the NCBI gene bank by blasting with *CPO* from *S. cerevisiae*. The knockout vector was constructed based on the homologous double-exchange strategy (Figure S1A,B). The upstream and downstream homologous fragments were amplified by PCR reaction and fused with the HPH fragment by touch-down PCR. A large amount of vector was amplified with a pair of nested primers. Then, the vector was introduced into PH-1 by PEG6000-mediated protoplast transformation. Protoplasts were obtained by digesting fresh mycelia with a mixture of enzymes (0.2 g snailase (Solarbio Life Science), 0.1 g lysozyme (Solarbio Life Science), and 0.1 g driselase (Sigma-Aldrich, Merck, Darmstadt, Germany) in a 20 mL 0.7 M NaCl solution for 2 h in a shaker (85 rpm, 30 °C). The detailed procedure for the protoplast transformation was based on a previous study [62]. And the knockout transformants were selected with hygromycin B and verified by PCR and Southern blot (Figure S1C–F).

The N-terminal GFP-tagged vector was constructed as follows: The GFP sequence (without stop codon) amplified from pYF11, the full length of the target gene (from ATG to stop codon), and the fragment of plasmid of pYF11 or pDL2 digested with *Xho* I were reconstructed by the 2xMultiF Seamless Assembly Mix (ABclonal Technology Co., Ltd., Wuhan, China). Expression of the fusion gene was initiated by the enhanced promoter RP27 on pYF11 or pDL2, with specific primers designed. The reconstructed plasmid was amplified by *Escherichia coli* DH5α (Sangon Biotech Co., Ltd., Shanghai, China). Colonies grown from LB plates containing 100 μg/mL ampicillin were verified by PCR and sequencing (Sangon Biotech). The resulting plasmid was then extracted with a plasmid extraction kit (OMEGA, Norcross, GA, USA) for subsequent transformation. And the resulting vectors were introduced into the strain to be transformed by protoplast transformation, as described above. Transformants were selected with hygromycin B or G-418. The GFP-tagged transformants were verified by PCR and fluorescence observation by the laser confocal scanning confocal microscope (Leica TCS SP8).

All the primers used in this study are listed in Table S1. Because several independent experiments showed that knockout of *FgCPO* or *FgFc* was lethal to *F. graminearum*, correct transformants were finally obtained on hemin-supplemented media. Hemin was also used in the following study.

### 4.3. Growth and Conidiation Determination

All strains were preincubated on CM plates containing 0.1 g/L hemin for 3 d at 25 °C in the dark. For the mycelial colony determination, CM or MM plates with/without 0.03 g/L hemin were prepared, and 5 mm diameter mycelial discs of each strain cut from the colony edge were transferred onto the center of the CM or MM plates and incubated for 3 d at 25 °C in the dark. For the growth rate test, the diameters of all the strains were recorded every 12 h. Hemin was added to the media after it was cooled below 60 °C during plate preparation, and this rule applied to all plate preparations. The mean of the two lengths of the colony in the vertical direction was taken as the diameter. The parent strain PH-1 added with hemin was used as the control. For conidiation determination, six 5 mm diameter mycelial discs cut from the colony edge were inoculated into 30 mL of MBB containing a range of hemin for conidia production in a shaker (175 rpm, 25 °C) for 7 d. Then, the conidia were filtered through three-layer lens wiping paper (Yutai, Hangzhou, China) and numbered with the hemocytometer (Marienfeld, Lauda-Königshofen, Germany) under the optical microscope (Nikon Instruments Inc., Tokyo, Japan) with a 10×/0.25 objective lens. Three replicates were performed for each treatment. All assays were repeated three times, independently.

### 4.4. Determination of Sensitivity to Fungicides and Various Stress Factors

To determine the sensitivity of the strains to fungicides, CM plates containing serial concentrations of tebuconazole (0, 0.03125, 0.0625, 0.125, 0.25, 0.5, 1, 2, and 4) or fludioxonil (0, 0.0125, 0.025, 0.05, 0.1, 0.2, 0.4, 0.8, and 1.6) were prepared, respectively. Meanwhile, 0.03 g/L hemin was added to the medium. Then, 5 mm diameter mycelial discs of each strain were inoculated into the center of the plates. After 3 d of incubation at 25 °C in the dark, the colony diameters were measured. The inhibition of each treatment was calculated using the formula: inhibition = (the diameter of the blank control—the diameter of the treated group)/(the diameter of the blank control—the diameter of mycelial discs). EC_50_ values were obtained based on the concentration and the corresponding inhibition. There were three replicates for each treatment. The parent strain PH-1 supplemented with hemin was used as the control, and the experiment was repeated three times independently.

To evaluate the effect of heme in regulating osmotic stress, cell wall stress, and oxidative stress, CM medium plates supplemented with NaCl (0.8 M, 1 M), KCl (0.8 M, 1 M), SDS (0.25 g/L), Congo Red (0.3 g/L), and H_2_O_2_ (5 mM, 10 mM) were prepared. H_2_O_2_ was added after the medium was cooled below 60 °C, and the other reagents were added before the media were sterilized. Then, 5 mm diameter mycelial discs were cut from the edge of the colony and transferred onto the center of the plates. After that, 0.03 g/L hemin was added to the medium to ensure the growth of the two mutants. After 3 d of incubation at 25 °C in the dark, colony diameters were measured. There were three replicates for each treatment. The parental strain PH-1 added with hemin was used as the control. The experiment was repeated three times, independently.

### 4.5. Lipid Droplet Staining and Glycerol Accumulation Detection

Mature conidia and mycelia incubated for 24 h were stained with 2.5 mg/mL of Nile Red (Macklin) to localize lipid droplets and observed under the laser confocal fluorescence microscope (Leica TCS SP8, Berlin, Germany). Fluorescence signals were detected with 514 nm excitation light, and the emission wavelength was set between 539 and 749 nm. Mycelia or conidia were observed with the objective of HC PL APO CS2 63×/1.4 OIL.

To determine the glycerol concentration, 8 mycelial discs were inoculated into 100 mL of liquid CM medium containing 0.03 g/L hemin and cultured in a shaker (175 rpm, 25 °C) for 3 d. Then, the mycelia were collected using a 200-mesh nylon net, washed with sterilized water, and the excess water was removed. Glycerol determination was measured using the glycerol kit (Applygen Technologies Inc., Beijing, China). Fresh mycelia were pulverized to powder with liquid nitrogen and incubated in the lysis buffer. Absorbance was detected using a SpectraMax M5 microplate reader (Molecular Devices, San Jose, CA, USA). A standard curve (r = 0.9999) was generated according to the kit instructions. All procedures were performed according to the instructions. There were three replicates for each treatment, and the experiment was repeated three times independently.

### 4.6. Determination of Fatty Acid Utilization

To test the utilization ability of fatty acid (50 mM), butyric acid (50 mM), caproic acid (50 mM), myristic acid (25 mM), palmitic acid (25 mM), or oleic acid (25 mM) as the sole carbon source, they were added separately into MM medium (pH 7.0) without a carbon source. Glucose (20 g/L) was used as a control carbon source. During plate preparation, 1% Tween 20 was added to promote the dissolution of fatty acids. Then, 5 mm mycelial discs were inoculated in the center of the plates. Each strain had 3 parallel replicates of each treatment. After 5 d of incubation at 25 °C in the dark, colony diameters were measured, and the relative colony diameter was calculated. The relative colony diameter = (the diameter of the treatment)/(the diameter of the glucose). The parent strain PH-1, supplemented with hemin, was used as the control. The experiment was repeated three times, independently.

### 4.7. RNA Extraction

Six 5 mm mycelial discs were inoculated into 30 mL of CM liquid medium supplemented with or without 0.03 g/L hemin and incubated in a shaker at 175 rpm at 25 °C for 3 d. The mycelia were then harvested, dried with absorbent paper, and rapidly frozen with liquid nitrogen. Total RNA was extracted with an RNA extraction kit (TIANGEN, Beijing, China). The integrity of RNA was identified by agarose gel electrophoresis, and the quality of RNA was evaluated by an ultraviolet spectrophotometer (Thermo Fisher Scientific).

### 4.8. Quantitative Real-Time PCR Analysis

The cDNA was synthesized by the HiScript II 1st Strand cDNA Synthesis Kit (+gDNA wiper) (Vazyme, Nanjing, China). The RT-qPCR reaction system was prepared with ChamQ SYBR qPCR Master Mix (Vazyme), and the reactions were performed on the Applied Biosystems 7500 (Applied Biosystems, Foster City, CA, USA). The relative expression level was analyzed by the 2^(−ΔΔC(t))^ method [63]. The procedure was performed according to the manufacturer’s instructions. GAPDH was used as an endogenous control. The parent strain PH-1 supplemented with hemin was used as the control.

### 4.9. Autophagy Detection

To determine whether heme affects autophagy, the vector containing GFP fused to the N-terminus of FgAtg8 (G-418 resistance) was induced into Δ*Fgcpo* or Δ*Fgfc*, respectively. Eight 10 mm diameter mycelial discs cut from the edge of the colony of each strain were crushed with a grinder (MM400, Retsch, Germany) and then added into 200 mL of CM liquid medium supplemented with 0.03 g/L hemin. The mycelia were incubated at 25 °C and 175 rpm in the dark for 24 h. Then, the mycelia were collected or shifted into MM-N medium to induce autophagy. The GFP signals were observed under the laser confocal fluorescence microscope (Leica TCS SP8) at 488 nm excitation light, and the emission light wavelength was set between 493 and 536 nm. In addition, the degradation of FgAtg8 was analyzed using Western blot. The protein extraction method was based on the previous study [64]. The primary antibody, anti-GFP rabbit polyclonal antibody GFP (300943), was purchased from Zenbio (Chengdu, China). The secondary antibodies (HRP-conjugated goat antirabbit IgG and HRP-conjugated goat antimouse IgG) were purchased from Sangon Biotech Co., Ltd. The GAPDH antibody was purchased from Proteintech (Wuhan, China). The ECL Plus Ultra-Sensitive Luminescent Liquid (Solarbio Life Science) was used for color development, and the FusionCapt Advance FX7 (VILBER BIO IMAGING, Marne-la-Vallée, France) was used for image capture. The grayscale values were analyzed with ImageJ (v. 1.51j8).

### 4.10. Statistical Analysis

All data were processed with the SIGMA-STAT Statistical Software Package (SPSS Science, version 25, International Business Machines Corporation, New York, NY, USA). Data from repeated experiments were used for the homogeneity of variance test, and when homogeneity of variance was equal (significance > 0.05), the data were then analyzed by one-way ANOVA and multiple comparisons based on the Fishers LSD test. When homogeneity of variance was not equal (significance < 0.05), Dunnett’s T3 test was used.

## 5. Conclusions

Our study shows that heme is crucial for the survival of *F. graminearum* and that heme is also involved in a variety of important biochemical processes in *F. graminearum*, including vegetative growth, asexual reproduction, glycerol accumulation, stress resistance, and fatty acid β-oxidation. In the future, we hope to screen novel small molecules that inhibit the activity of FgCpo or FgFc based on interspecies differences and thus develop new fungicides.

## Figures and Tables

**Table 1:** Sensitivities of the parent strain and the mutants to tebuconazole and fludioxonil.

Strains	Tebuconazole		Fludioxonil	
	EC_50_ (μg/mL) ^z^	MIC (μg/mL)	EC_50_ (μg/mL) ^z^	MIC (μg/mL)
PH-1	0.0422 ± 0.0030 c	2	0.0313 ± 0.0036 d	0.4
PH-1 + hemin	0.0616 ± 0.0044 a	2	0.1136 ± 0.0047 a	1.6
Δ*Fgcpo* + hemin	0.0475 ± 0.0037 c	0.5	0.0479 ± 0.0032 b	0.4
Δ*Fgfc* + hemin	0.0554 ± 0.0016 b	0.5	0.0379 ± 0.0009 c	0.4

^z^ The same letters following the mean in the same column denote that they are not significantly different based on a one-way ANOVA followed by Fisher’s LSD test at *p* = 0.05.

## Data Availability

The data that support the findings of this study are available from the corresponding author upon reasonable request.

## Supplementary Materials

The supporting information can be downloaded at: https://www.mdpi.com/article/10.3390/ijms25105268/s1.
