# Supplementary material for: Crucial Involvement of Heme Biosynthesis in Vegetative Growth, Development, Stress Response, and Fungicide Sensitivity of Fusarium graminearum
Source: Int J Mol Sci. 2024 May 12;25(10):5268. doi: 10.3390/ijms25105268 (PMC11120706; doi:10.3390/ijms25105268)
Supplement: Supplementary file 1 [file ijms-25-05268-s001.zip › Figure S1.pdf]

Supplementary materials

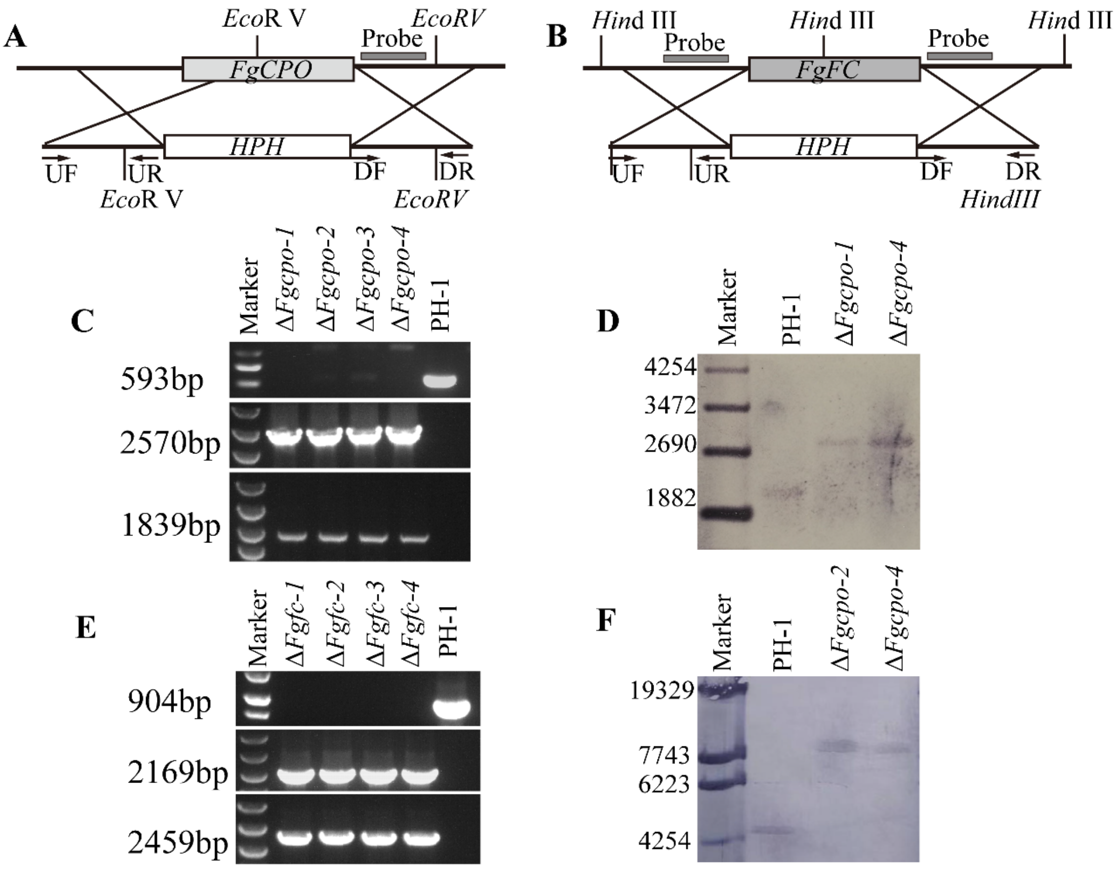

Figure S1. Vector construction strategy and verification of mutants. The knockout vector was constructed based on the principle of homologous double exchange. Primers used for amplifying the homology fragment were indicated in the figure. (A) Schematic representation of Δ*Fgcpo* vector construction. (B) Schematic representation of Δ*Fgfc* vector construction. (C) PCR verification of Δ*Fgcpo*. The target gene fragment, upstream and downstream were identified separately. (D) Southern blot verification of Δ*Fgcpo.* Genomic DNA was digested with *Eco*R V and separated by TAE gel electrophoresis. The probe was binding to the downstream of FgCPO (E) PCR verification of Δ*Fgfc.* The target gene fragment, upstream and downstream were identified separately. (F) Southern blot verification of Δ*Fgfc.* Genomic DNA was digested with *Hin*d III and separated by TAE gel electrophoresis. The probe was binding to the upstream of FgFC.
