# Supplementary material for: Crucial Involvement of Heme Biosynthesis in Vegetative Growth, Development, Stress Response, and Fungicide Sensitivity of Fusarium graminearum
Source: Int J Mol Sci. 2024 May 12;25(10):5268. doi: 10.3390/ijms25105268 (PMC11120706; doi:10.3390/ijms25105268)
Supplement: Supplementary file 1 [file ijms-25-05268-s001.zip › Table S1.pdf]

Table S1 Primers used in this study

Primer names	Sequence (5'-3')	Annotation
FgCPO-UF	TGCTTGTAAGTGCCGTATTG	Primers to amplify FgCPO upstream fragment
FgCPO-UR	AGCCAGCCAACAGCTCCCTCATCGCAGTCGCATGGG	
FgCPO-DF	CGCAAACCGCCTCTCCCCAAGCCATTAATAACTTTC	Primers to amplify FgCPO downstream fragment
FgCPO-DR	CATCACAGCCTTCCACGA	
FgCPO-NF	TCAGGCTGCTTCAGTAAAT	Nested primers to amplify the FgCPO vector
FgCPO-NR	AAGCCAAGGAAGGTGGTGAGA	
HPH-F	GGGAGCTGTTGGCTGGCT	Primers to amplify the fragment of hygromycin
HPH-R	GGGGAGAGGCGGTTTGCG	
Y-FgCPO-F	CCGTAAGGACGAGTGGGA	Verification of FgCPO fragment
Y-FgCPO-R	CGATGATGGGAATGTAGGAG	
Y-CPOUF	TACATACGGGAATACGAT	Verification of FgCPO upstream binding region
Y-CPOUR	AGGGCAAAGGAATAGA	
Y-CPODF	CCGCCGCTACTGCTACAA	Verification of FgCPO downstream binding region
Y-CPODR	CAACCACCGCACGAACAT	
Pro-CPO-F	AGCCTTGTGATAATGGAGA	Probe for Southern blot verification of Δ*Fgcpo*
Pro-CPO-R	GAGATGAACGATGTAGCG	
GFP-F	GTAGGAACCCAATCTTCAAAATGGTGAGCAAGGGCGAG	Primers to amplify the full length of GFP
GFP-R	CTTGTACAGCTCGTCCATG	
GFP-FgCPO-F	TTTCGTAGGAACCCAATCTTCAAAATGGCTGCCAGACAGCCAGC	Primers to amplify the full length of FgCpo for GFP-labeled
GFP-FgCPO--R	CACCACCCCGGTGAACAGCTCCTCGCCCTTGCTCACGACCCACTCCTTGGGATC	
Y-GFP-FgCPO-F	CAGTGCTTCAGCCGCTAC	Verification of GFP-FgCPO vector
Y-GFP-FgCO-R	TCTTGGAGAACGCAGGTC	
FgFCUF	TCTGGTAATGGGAAATAGG	Primers to amplify FgFC upstream fragment
FgFCUR	AGCCAGCCAACAGCTCCCTTGAAAGTGGTGGGAAAG	
FgFCDF	CGCAAACCGCCTCTCCCCTCATACCTTTACGGGAGA	Primers to amplify FgFC downstream fragment
FgFCDR	AGTTGCCATCGTGCTA	
FgFCNF	ATTTGCCGCTTTGGTC	Nested primers to amplify the FgFC vector
FgFCNR	TGTTTCCTTGGTGGGT	
Y-FgFC-F	GCTCAAAGGCGGTATCTG	Verification of FgFC fragment

Primer names	Sequence (5'-3')	Annotation
Y-FgFC-R	TCGGTAAGGGTTGGAAAA	
YZ-FgFCUF	ATTGTGGCGGGAACTA	Verification of FgFC upstream binding region
YZ-FgFCUR	AGGGCAAAGGAATAGA	
YZ-FgFCDF	CGCTCGTCTGGCTAAG	Verification of FgFC downstream binding region
YZ-FgFCDR	ATTGTGGCGGGAACTA	
Pro-FC-F	CTCGGCTGTCGCCTGATT	Probe for Southern blot verification of Δ*Fgfc*
Pro-FC-R	CACCTAGCTCGCGTTTGC	
FgFCGFP-F	GCGAATTGGGTACTCAAATTGGTTATGGCTTCGAGAATATCTAC	Primers to amplify the full length of FgFC for GFP-labeled
FgFCGFP-R	CAGCTCCTCGCCCTTGCTCACTTACGCTAGGCCAGCCTGCTG	
Y-GFP-FgFC-F	AGCAGAAGAACGGCATCA	Verification of GFP-FgFC vector
Y-GFP-FgFC-R	TCGGTAAGGGTTGGAAAA	
Q-FgCYP51AF	CTTCTCCAGAACGTCGTCAA	Quantitative primers of FgCYP51A
Q-FgCYP51AR	CTTCAGTGTCAACCTCCTCC	
Q-FgCYP51BF	TGCTCCTATCCACTCCA	Quantitative primers of FgCYP51B
Q-FgCYP51BR	GCTGAACCCTTGCTGAC	
Q-FgCYP51CF	ATCGTCCACCCGTTGTAT	Quantitative primers of FgCYP51C
Q-FgCYP51CR	GCAGTCGCCGTATTTATCT	
Q-FgSSK2F	AGGAGACGGCGACTGA	Quantitative primers of -FgHOG1
Q-FgSSK2R	TCGGGTGGGAATGAAA	
Q-FgPBS2F	CTGGGCAAGGGAAACTAC	Quantitative primers of -FgHOG1
Q-FgPBS2R	ACTGGTGAAGGGTCTGATT	
Q-FgHOG1F	CCATCGCCAGTGAGAACA	Quantitative primers of -FgHOG1
Q-FgHOG1R	TAGGGAGAAAGGTAGTCGTG	
Q-GAPDH-F	GGGTGACCTCAAGGGTGTTC	Quantitative primers of GAPDH
Q-GAPDH-R	AGTAACCCCACTCGTTGTCG	
